# Ethical considerations for DNA testing as a proxy for nationality

**DOI:** 10.1080/11287462.2021.1896454

**Published:** 2021-03-25

**Authors:** Valedie Oray, Sara H. Katsanis

**Affiliations:** a Initiative for Science and Society, Duke University, Durham, NC, USA; b Mary Ann & J Milburn Smith Child Health Research, Outreach, and Advocacy Center, Ann & Robert H Lurie Children’s Hospital of Chicago, Chicago, IL, USA; c Department of Pediatrics, Feinberg School of Medicine, Northwestern University, Chicago, IL, USA

**Keywords:** Statelessness, DNA testing, ancestry, immigration, border policy

## Abstract

As nations strengthen borders and restrict refugee admissions, national security officials are screening for fraudulent nationality claims. One tool to investigate nationality claims is DNA testing, either for claimed relationships or for ancestral origins. At the same time, the plight of global statelessness leaves millions without documentation of their nationality, and DNA testing might be the only recourse to provide evidence of heritage or relationships. DNA testing has been used sparsely to date to determine ancestral origin as a proxy for nationality but could increase as border controls tighten. Given the historic lessons in eugenics and the potential for misuse of personal genetic information, it is essential to consider the ethical parameters in order to guide the implementation of genetic data for such purposes. Here, we break down examples of the use of DNA testing for nationality, and the risks and benefits of genetic testing for this purpose. Important ethical considerations discussed include (1) empowerment of stateless individuals with evidence for citizenship proceedings; (2) imprecise correlation between genetic heritage and nationality; (3) effective protection of state interests; and (4) practicalities of DNA testing.

## Introduction

Certain rights are associated with having a nationality, including the ability to access economic and social benefits, to vote, and to freely enter and leave the state in which they reside (Vlieks et al., 2017). In 1948, the United Nations General Assembly drafted within “The Universal Declaration of Human Rights” provisions that “everyone has the right to a nationality,” and that “no one shall be arbitrarily deprived of his nationality nor denied the right to change his nationality” (*Universal Declaration of Human Rights*, 1948). This declaration of nationality as a human right led to efforts to provide state citizenship to stateless individuals (United Nations High Commissioner for Refugees, 1954, 2014). The processes for granting a person with nationality, however, is the responsibility of each state. Many states grant citizenship to individuals as a privilege rather than an entitlement (Singer & Singer, 2010), leaving varyingly complicated, exclusionary, or ineffective policies around the world that render some people stateless (Kingston, 2013).

The United Nations High Commissioner for Refugees estimates that 10 million people are stateless, which is lacking documentation of citizenship to any country (United Nations High Commissioner for Refugees, 2015). Whether a person is *de facto* stateless (having nationality, but excluded by the state) or *de jure* stateless (lacking nationality and papers), the UNHCR has struggled to develop approaches to provide evidence of statehood for several decades (Foster & Lambert, 2016; Lori, 2017). Stateless persons without any form of formal documentation, such as a birth certificate or state identification, face barriers trying to gain access to state privileges attached to their nationality. Being a stateless individual poses issues when attempting to access human rights, including the ability to obtain basic healthcare, employment, and education (Vlieks et al., 2017). Citizen privileges, including having a job, getting married, or opening a bank account, are also impossible for stateless people to obtain (United Nations High Commissioner for Refugees, 2014). The denial of state membership also threatens personal security. Stateless persons are vulnerable to economic, social, and political threats without any ability to protect themselves or for the state to mitigate these risks (Sokoloff & Lewis, 2005). They often face the risk of being expelled from their residence based on their lack of citizenship (van Waas & Jaghai, 2018). Statelessness is particularly acute for migrants and refugees that leave their countries of origin without traveling papers.

States have valid concerns in screening for fraudulent identities among refugees and stateless persons that could conceivably seek refugee status, seek immigration benefits or commit acts of harm or terrorism, based on deception (Grütters & Zwaan, 2010; Rudner, 2008). The United Kingdom has established policies for handling disputed and/or doubted nationality claims (UK Home Office, 2017). In 2009, the United Kingdom attempted to pilot an effort to incorporate biogeographic DNA testing and isotope analysis as a proxy for nationality for investigating suspected fraud in the asylum petition process (UK Home Office, 2010). In the United States, a 2008 pilot of relationship DNA testing of 3,000 migrants from a variety of African countries found that upwards of 80% of cases were considered potentially fraudulent when families refused to subject to DNA testing (Esbenshade, 2010). Whether or not refusal to undergo DNA testing was due to deception, misunderstanding, or fear of providing DNA was not documented (Kim, 2011). In 2018, the Canadian Border Services Agency reportedly used ancestry testing to verify the nationality of a Liberian, and speculated fraudulent identity when the results indicated a different place of origin (Hopkins, 2018; Khandaker, 2018).

The use of DNA testing is common enough in immigration proceedings for verifying family claims in several countries (Heinemann & Lemke, 2013; Heinemann et al., 2013; Holland, 2011; Katsanis & Kim, 2014; Kim, 2011; Lee & Voigt, 2020). The UNHCR supports the use of DNA testing for family relationships only in cases with strong indications of fraud (United Nations High Commissioner for Refugees, 2012). Typically, DNA testing involves a comparison of a parent and child or similarly closely related family members to verify a biological relationship claim. DNA testing is becoming more prevalent in immigration proceedings, and as we acknowledge that ethical concerns remain for *relationship DNA testing* in immigration, we are interested in exploring the potential use case of *ancestry DNA testing* as a proxy for nationality in future immigration proceedings and how the relationship and ancestry DNA testing can be applied to provide evidence of statehood for stateless persons. While this is a hypothetical scenario at this time, the prior use cases of biogeographical information in the UK and Canada underscore the realistic potential implementation of this approach to screening migrants. The analysis and discussion here are intended to guide further conversations to inform policy options for protecting stateless persons and maximizing rights to both nationality and migration.

## DNA testing for nationality

Similar principles for immigration DNA testing could apply for providing evidence of nationality. For example, relationship testing of a parent and child, which is common for visa petitions, can also be applied for demonstrating relatedness of a stateless person to a person with state recognition (Flaim, 2017). The other way to use DNA as evidence of nationality is the use of ancestral DNA markers. This type of test is not looking at the relatedness of two individuals, but rather, provides genetic clues as to a person's biological heritage. Both tests are important to consider in nationality claims, but also have different ethical considerations, as described in detail below. Table 1 outlines examples of contexts where DNA testing was used or considered as evidence of a person's nationality.

**Table 1.** Case examples of DNA testing used as evidence of nationality or statehood.

Scenario	Narrative
Relationship testing to establish nationality documents for historically excluded populations	Plan International began a project in the early 2010s to sponsor relationship DNA testing for families and children in the northern hills of Thailand for citizenship (Flaim, 2017) The subsidized, state-sponsored DNA testing provided evidence of familial ties in Thailand for people to provide evidence that they are of Thai descent No formal process was set by Thailand to use genetic identity for citizenship purpose; importance weighed differently between law officials (Flaim, 2017)
Biogeographical ancestry to determine country of origin in a migrant stateless person	A stateless individual who emigrated from a country in Southeast Asia as a minor ten years prior took refuge in a European country, but could not gain formal refugee status The person did not have formal documentation of citizenship from any country, nor did they know their own origin for certain, given the death of their biological mother at a young age The immigration attorney in the European country sought a genomic ancestry DNA test in 2018 to examine genetic markers that might indicate ethnicity, and to discriminate between a country that could classify the migrant as a refugee This case is ongoing. It is unknown how or whether the genomic results will be a factor in the legal proceedings nor how a court would interpret the results of any genomic report (personal communication, Professor Katsanis)
Biogeographical ancestry to limit refugee petitions to certain nationalities	In 2009 the United Kingdom Border Agency proposed “The Human Provenance Project” to use ancestry genetic tests on African asylum-seekers to verify Somalian nationalities (UK Home Office, 2010) DNA testing was proposed to examine genomic markers to differentiate Somalian petitioners from other nationalities (e.g. Nigerian, Liberian), in combination with isotope ratios in hair and fingernails to establish where migrant petitioners had most recently lived (Hill & Henderson, 2011; Travis, 2009) The pilot program was canceled after widespread criticism (Travis, 2009)
	In 2018, the Canadian Border Services Agency (CBSA) used an ancestry DNA test to verify nationality of a migrant (Hopkins, 2018; Khandaker, 2018). The migrant stated that he is Liberian, but the DNA test results, when compared to others in the database indicated relatives from Nigeria, causing the CBSA to speculate he is using a fraudulent identity (Hopkins, 2018) The ancestry DNA company claimed that they were unaware of the CBSA's use of their ancestry testing for this case (Khandaker, 2018)

### Documentation of nationality

Proof of identity (POI) at a border includes three main attributes: biometric (e.g. fingerprints), attributed (e.g. a full name), and biographical (e.g. education or employment history) (Katsanis & Kim, 2014). Because of the ease of creating false documents, the reliance on attributed and biographical data alone is waning (Jamieson et al., 2008). Biometrics are increasingly included in POI documentation and considered in screening immigration petitions.

Refugees seeking status in a new country are required to provide documentation that includes their name, date and place of birth, and their current residency. Typically, the best form of identification is state identification, a birth certificate, a driver's license, or a passport; however, in some instances, these documents might not be available, particularly when refugees are fleeing out of danger. Other acceptable documents can include a marriage certificate, any medical records, or admission into a school or daycare (United Nations High Commissioner for Refugees, 1984). In some instances, an individual's country of origin will be contacted directly to seek appropriate documentation regarding the person. Oftentimes a state will not be able to comply or provide the necessary proof of citizenship. Without documentation, an individual can find significant delays in their immigration petitions.

There are unconventional methods for providing evidence of identity that can be useful in some circumstances. For example, the software can analyze different nuances in English handwritings of the same text to detect the nationality of an individual with a 53.66% accuracy (Al Maadeed & Hassaine, 2014). Similarly, different character forms of Arabic handwriting can associate an individual with a specific nationality with up to 83% accuracy (Al-Hadhrami et al., 2015). Some countries determine a person's place of origin based on their dialect through language analysis for the determination of origin (LADO) (Craig, 2012; Wilson & Foulkes, 2014). Identifying an accurate place of origin based on linguistic patterns can be difficult, especially if an individual comes from an area with numerous different dialectal influences or may find it hard to speak in their home dialect to a government representative (Craig, 2012; McNamara et al., 2016). Furthermore, no standardized procedure is in place for conducting LADO, so the final determinations of one's nationality might not be reliable (Wilson & Foulkes, 2014). Absent these tools, there could be opportunities to use photographs or scraps of evidence like news articles or receipts that can help verify one's identity or their relationship to others.

### DNA testing in immigration

DNA testing is used in a variety of immigration contexts, including family reunification visa petitions and screening of migrants to verify relationship claims (Katsanis & Kim, 2014; Travis, 2009; Wagner et al., 2019). Over twenty countries use parental DNA testing in some capacity for immigration cases where families are unable to provide documentation of biological relationships (European Migration Network, 2012). In Germany, for instance, the Federal Foreign Office determined that over forty countries do not have a proper system of identification to verify familial relationships through paper documents, so families are encouraged to provide genetic information to verify relationships in order to immigrate with their children or relatives (Lee & Voigt, 2020).

The United States encourages DNA testing for parental relationships for families who are attempting to transmit citizenship to their children who are abroad when formal documentation is not available. DNA testing also is occasionally requested to verify biological relationship claims when individuals petition for a visa to the United States (U.S. Department of State, 2021). The recent influx of family unit migration at the United States-Mexico border prompted new policies to use DNA tests to detect fraudulent familial claims and potentially child trafficking (Farahany et al., 2019; U.S. Department of Homeland Security, 2019; Wagner et al., 2019). The United States also recently began collecting DNA from migrant detainees for the federal criminal DNA database (“DNA sample collection from immigration detainees,” 2020).

### Relationship DNA testing

Different DNA tests can provide different kinds of evidence for biological relationships. Short tandem repeat (STR) DNA analyses examine a set of certain locations in DNA that tend to be highly variable from one person to the next. By looking at a specific set of such markers, the numbers of copies found at each STR location comprise a unique genetic profile for an individual. This type of STR analysis is commonly used for kinship analysis (e.g. paternity testing) and can indicate the likelihoods of biological relationships of immediate family members, including siblings (Wenk, 2004). Autosomal STR tests for kinship are fairly accurate for determining the likelihood of a parent and child being related, and X- and Y-STRs can supplement these data (Diegoli, 2015). Male heredity can be traced through Y-chromosome STRs since the Y-chromosome is inherited from a father to his son (Kayser, 2017), whereas X-chromosomes are in both males and females. Y-chromosome DNA also has high conservation between the father and male offspring, so Y-STR testing is also considered reliable in terms of accurate detection of the variants at each marker (Kayser, 2017). Determining full siblings and grandparents using STRs is also fairly reliable, but half-siblings and avuncular relationships have less reliability since they only shared 25% of their genetic makeup between one another (Katsanis & Kim, 2014; Zhang et al., 2020).

Mitochondrial DNA testing analyzes the genetic sequence variants found in the mitochondrial DNA, which is inherited from the mother to her offspring, and can indicate maternal heritage (Wenk, 2004). Mitochondrial DNA sequences are reliable due to mtDNA's high conservation from one generation to the next (Amorim et al., 2019). Both mitochondrial and Y-STRs are imprecise for determining exact relationships but are good indicators of distant relationships along direct maternal or paternal lines.

Autosomal single nucleotide polymorphisms (SNPs) are used to look at hundreds of thousands of variable locations in the genome. A single SNPs is less variable than a single STR but informative in revealing patterns in the inheritance of thousands of SNP variants. Like STRs, SNPs can reveal close biological relationships (e.g. parent–child), but also distant relationships like cousins and great-grandparents. The more distant the relatives are the less precise the comparisons will be. SNPs are commonly used in consumer genomics and in genomic research to test for variants that can guide risk for health conditions and other traits (Campa et al., 2011; Kidd, Pakstis, et al., 2014; Mehta et al., 2017; Pavan & Sturm, 2019).

### Biogeographical ancestry DNA testing

Genomic ancestry tests utilize autosomal SNPs to hypothesize ancestral origins of individuals of both recent and ancient prior generations (Jorde & Bamshad, 2020). Certain SNPs that are conserved among discrete populations are called ancestry informative markers (AIMs) (Kidd, Speed, et al., 2014; Phillips, 2015). The pattern of AIMs can be used to calculate the likelihood of one's ancestral place(s) of origin, providing clues to a person's genetic background and their heritage (Kosoy et al., 2009). Consumer interest in genomic ancestry has driven this technology and the growth of a commercial industry for ancestry DNA testing. In addition, several examples in research highlight how AIMs can reveal genetic origins (Kampuansai et al., 2017; Kerminen et al., 2017; Muzzio et al., 2018).

Technically, the typing of individual SNPs is generally accurate, but the interpretation of the patterns of thousands of typed SNPs can lead to varied results. Inconsistencies come from the different sets of markers from different testing companies and the computational differences in evaluating the patterns of inheritance. The SNPs that companies analyze in a genetic sequence varies between tests. The computations are reliant on control population data that can be highly different from one study to the next, especially for more discrete populations (e.g. Welch vs. English). Consumer genomics companies lack a formal system to share data and control groups, and lack of consistent criteria leads to differences in calculations of DNA test results (Committee on Genetics, 2017). As a result, companies can derive different conclusions about a person's ancestry, even when looking at the same genetic markers (Imai et al., 2011).

## Ethical considerations

The use of new technologies with high stakes, potentially for individuals, families, and governments, demands an assessment of the potential ethical issues that could arise with the technology. Formal studies and evaluations of the effects of these technologies are essential, including case studies, interviews, outcomes research, and comparisons to other technologies or approaches. In this early stage of using DNA as evidence for nationality, with few cases to date, but with high potential for its use, here we frame the ethical considerations as bodies debate the practicalities and policies for implementing genetic information into their procedures. The following considerations are not recommendations but are intended to provide a foundation for debate on the use of relationship DNA and ancestry DNA tests to provide evidence of nationality.

### Conflating genetic heritage and nationality

Genomics advances have improved the ability of genetic data to reliably predict the composition of a person's ancestral heritage in many circumstances; however, measurement of ancestry is not equivalent to nationality. National borders are drawn upon political lines, whereas biological ancestry predicts biological heritage among certain populations. Populations, of course, move and intermingle, so equating a genetic ancestry test result to nationality is erroneous. Similarly, genetic ancestry is not equivalent to race, as the race is a social construct, not a biological one.

**Figure 1.**
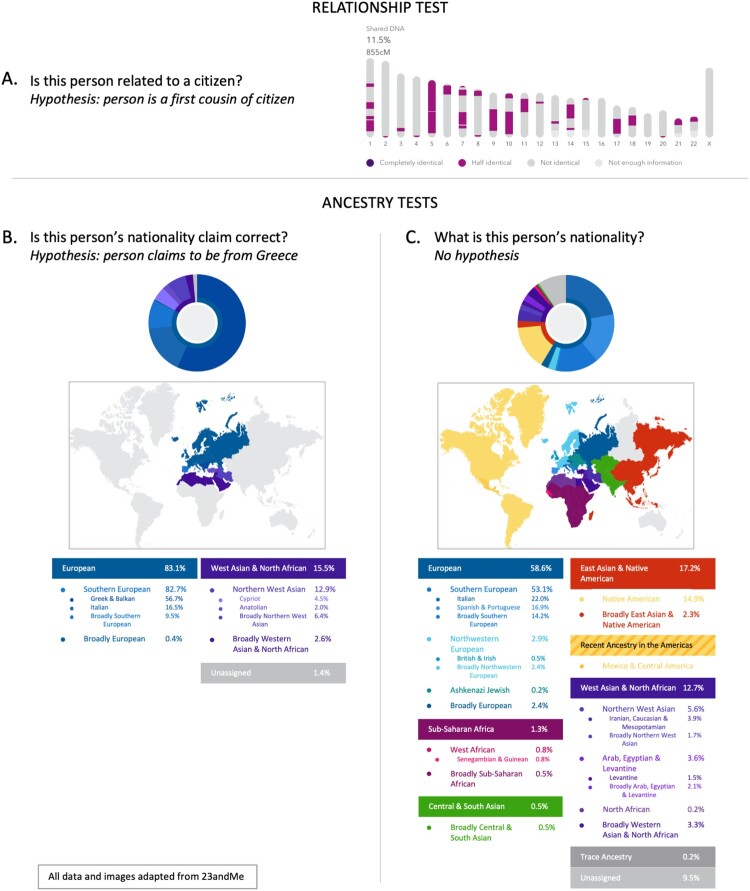
DNA testing can provide data to support relationship and ancestry claims. All results and images adapted from 23andMe genetic test results; permission was granted from individual data providers to publish anonymized results. (A) A relationship test will examine the commonalities between the DNA of two individuals, with the scientific assumption that no two people will share more than one part of their DNA except through coincidence. This comparison shows the overlapping shared regions of DNA among first cousins. The data shown would support such a relationship claim. (B) and (C) show ancestry test results for two individuals. In (B) the person hypothetically could claim to have Greek nationality. The data supports this claim, although other ethnicities are clearly apparent in this individual's DNA. The person could be from Greece or could be an American with Greek heritage. In (C) the person's genetic heredity exemplifies a person with a diverse genetic background. This person could claim heritage from many places, so establishing a single nationality would be challenging.

Relationship DNA testing can provide clues to verify family relationships, but a lack of a biological relationship does not negate a valid family structure (Dove, 2013). In the same way, a biogeographic DNA test can provide clues to a person's place of origin, but a lack of ancestral DNA from a certain region does not negate a valid nationality. A person's genetic makeup might be useful to predict ancestral heritage; however, it cannot accurately determine the country that they were born in (see Figure 1). Predicting ancestry is even more challenging for persons with mixed ancestral background. Interpretation of genetic ancestry could indicate a person is descended from multiple regions, and therefore ambiguous for nationality purposes. If a person claims that they are from a specific geographic region, and this region appears on their genetic test results as only a portion of their heritage, then it is to the discretion of the state to determine whether the DNA tests are sufficient evidence that the person is from that region. This sort of discretion can lead to prejudice and bias, a serious risk for the implementation of quotas or arbitrary “one drop” policies for inclusion (Jordan, 2014). If the state places an “ancestral percentage” quota, then it could lead to populations or individuals being barred from state membership based on their DNA tests results.

Nationalism based on genetic ancestry is a valid concern. If ancestry DNA tests to verify a migrant's place of origin is required as a condition of state membership, then nationalist tendencies are inevitable. The societal definition of nationality could shift to include that genetic ancestry must reflect one's place of origin, leading certain population segments to claim themselves as “true” citizens of a state based on their genetic ancestry and discriminate against those whose genetic backgrounds differ.

Our shared, global, and social history of eugenics and racial bias demonstrates the power and peril of discrimination against others based on genetic makeup (Friedmann, 1997; Provine, 1973). Conflating nationality with genetic ancestry could encourage modern-era eugenics by promoting certain ancestral lines or by stigmatizing other lines. False conflation could prompt policy changes to make genetic testing a more prominent aspect of immigration proceedings, as well. For example, when the United Kingdom piloted DNA testing for Somalian ancestry (UK Home Office, 2010), the intention was to detect fraud, but the reality was a discriminatory screening policy for black-skinned immigrants that would have stigmatized Somalians lacking “pure” ancestry (Hill & Henderson, 2011; Holland, 2011; Staff, 2017).

### Use of DNA as a tool to promote migration versus denying admission

In circumstances where a person lacks paper documentation for their place of origin and necessitate evidence of nationality for a petition for migration, ancestry DNA testing might provide valid evidence of their ancestral origins. Using ancestry DNA testing to identify an individual when no other evidence is available could advance a person's state petition. Certain markers exist in genetic code that can predict the place of origin for an individual (Kampuansai et al., 2017; Kerminen et al., 2017; Muzzio et al., 2018). Such genetic data could supplement other information, such as oral narrative and linguistics to assist in determining nationality. Relationship tests can similarly be valuable in providing concrete data to support a petition if one individual in the relationship can also prove their nationality. In these instances, the state has the ability to overcome requirements to prove documented nationality for admission and consider stateless individuals for refuge using biogeographical or relationship DNA results.

Because there is a risk that genetic heritage may not correlate to nationality, when biogeographic DNA results do not support a migrant's narrative, these results should be nullified and not be used to deny admission. Both ancestry DNA tests and relationship DNA tests might reveal information that is different from a person's narrative, or in fact, a surprise to the person. The DNA tests could reveal misattributed parentage, for example, which is relatively common in immigration petitions. Ancestry DNA tests might reveal heritage that is different from the expectations of the petition, as well. If DNA results do not support a person's narrative, then the other aspects of a migrant's petition and background must be considered before making a decision on their admission.

State-driven ancestry DNA testing to exclude particular populations from admission into a country would be counter-productive, discriminatory, and would likely lead to fraudulent testing paradigms. Since ancestry and relationship DNA tests both can reveal results inconsistent with a petitioner's claims, DNA testing would not be valid enough for systematic screening. Genetic tests as a tool for revealing fraudulent petitions could be attractive, but the high false positive and false negative rates in verifying a nationality render the tool inept.

### Protecting a state and its people

Fraudulent claims in refugee petitions is a real concern. Reported fraud in U.S. refugee and immigration programs has motivated the requirement of relationship DNA testing to verify claims in family reunification refugee petitions (Esbenshade, 2010; Holland, 2011; Katsanis & Kim, 2014). Migrants can lie about their identities in order to take advantage of a country's protection and resources; however, documented examples of fraud are a small proportion of cases (e.g. about 100 cases out of 230,000 in a 2017 pilot in German) (European Migration Network, 2012; Thüer et al., 2018). When a person has no ability to verify their native country, they are in a position where they could lie about their place of origin, whether for personal advantage or out of desperation. The state does have an obligation to verify migrant identities to protect its citizens from fraudulent individuals.

### Empowerment of individual migrants

Migrants who cannot demonstrate their place of origin are significantly disadvantaged when engaging with government officials that can provide basic state protections and privileges. Stateless persons often are burdened with providing evidence that they are stateless in immigration proceedings through several interviews and application scrutiny, lengthening petition processing. Refugee processing can also be a subjective process, vulnerable to officials’ predisposed prejudices and disbelief if migrants are unable to prove their persecution (Souter, 2011). DNA testing can alleviate these issues in some circumstances by providing scientific data to support their claims, which can reduce subjective speculation regarding one's narrative about their place of origin. DNA test results can be empowering for a stateless person. If the DNA data can provide support for a claim, they could acquire POI to support a state membership petition.

Whether DNA tests are used for inclusion or exclusion, the provision must be accompanied by a robust consent process. It is necessary to develop an easily comprehendible consent process that will accommodate all persons, regardless of their linguistic backgrounds and education levels. Voluntary consent is impossible when individuals petitioning for refugee aid or citizenship feel intense pressure to comply with the demands of immigration and government officials. Nevertheless, information provision is essential to outline the risks and benefits of genetic testing. Potential testing subjects should be informed about how their genetic ancestral or relationship data will be used, including whether or not their DNA data will be used for other purposes, like being archived for future crimes. They should also be informed of the potential benefits that can come from pursuing a DNA test, such as if DNA test results can expedite their petition.

In the case of minors, consent for DNA testing would normally be deferred to their parents or legal guardians. However, if biological parents are available, then ancestry DNA tests can be performed on them rather than the child since the test would reflect similar results, and to avoid undue risk of misattributed parentage. In cases of unaccompanied minors entering a country and requesting refugee status, consent would have to rest with a minors’ temporary guardian, so care should be taken as to the importance of the test to the overall petition process.

Following DNA testing, sharing the results with the test subject is essential. If DNA testing reveals incidental findings, like misattributed parentage or unexpected ethnicities, then a properly trained counselor should be involved in explaining the results. Sharing this data, however challenging, is important for the communication of how their genetic heritage affects their refugee or citizenship petition. Ancestry DNA tests can cause individuals to question their self-identity if they do not understand the results or to change their racial or ethnic identity based on their results (Foeman et al., 2015; Roth & Ivemark, 2018), so it is important to explain that one's genetic makeup is not necessarily indicative of their place of origin, their family's heritage, or the definition of family. Furthermore, it is important for immigration and government officials to provide transparency on how DNA test results affect petitions.

### Practicalities of use

DNA testing for ancestry or relationships can be a burden on financial resources to the state, and can be complicated from the collection process to receiving the results weeks later. Alternative methods of proving nationality can be less controversial if they have yielded accurate results, can be more resource-efficient, and does not conflate place of origin with genetic ancestry. Furthermore, practical matters limit the value of DNA testing in comparison to other screening measures. First, DNA tests must be reliable. It is important to consider which genetic testing methodology will be used. For instance, different consumer genomics ancestry tests yield varying results on the genetic makeup of an individual (Imai et al., 2011). While relationship tests have been accepted into immigration practices and undergo some degree of scrutiny through the American Association of Blood Banks (Yates, 2005) ancestry DNA tests have no oversight. There is no system to accredit who is an ancestry DNA test expert, nor standards for population controls or reporting. In individual cases, it might be valuable to consult a population geneticist with expertise in a particular population, rather than relying on a consumer genomic ancestry test report.

Second, if the test is to be used in a legal petition for citizenship, then a chain-of-custody process is necessary to verify that the DNA test results correspond to the petitioner. This would maintain accountability for the testing process, ensure that the correct results are presented, and ensure confidentiality of the data.

Third, it is important for an ancestry DNA test to be conducted by a laboratory that is blind to the expected outcome to prevent laboratory personnel prejudices based on a name or physical description or appearance.

## Conclusions

Statelessness affects millions of individuals worldwide, disadvantaging access to basic privileges such as quality healthcare, welfare from the government, a proper education, the ability to receive a job, and other aspects of a daily life that a citizen takes for granted (United Nations High Commissioner for Refugees, 2015). Stateless persons who wish to seek citizenship (or refugee status if they flee their home country) are unable to progress their petitions if they lack credible documentation of identity. The concerns derived from the few examples in Table 1, in addition to the uncertain accuracy of DNA tests proving ancestry, highlight a need for general public discussion on the implications of DNA testing for nationality. The ethical considerations presented here will aid in making informed decisions on the use of genetic testing.

DNA is a unique identifier that every individual has, so is an invaluable tool for documenting identity and verifying biological relationships. The technological advancements make this tool an imprecise but informative tool also for genetic ancestry. With fraud a common concern among government officials processing refugee petitions and asylum claims, the reliance on scientific evidence to support or refute a petition is tempting. The imprecision renders the tool no more effective than reviewing paper documents. Unlike paper documents, genetic data is difficult to fake. For this reason, utilizing DNA testing in cases to support an individual might be valuable as additional evidence of nationality, and to empower displaced persons. For individuals who do not have any additional form of documentation to prove their place of origin, DNA testing could be a critical component in their immigration or legal proceedings. However, the use of ancestry DNA testing broadly as a precondition for state memberships will lead to additional ethical issues, including stigmatization, discrimination, bias, and potentially eugenics. For refugees who might have other forms of legitimate identification, genetic information cannot be a justified addition to their petition.

People with an ambiguous genetic heritage might not be able to verify that they are from the country they identify with using their test results; however, this should not implicate the petition to be fraudulent. Instead, this should indicate that an individual's genetic ancestry is not the best tool for verifying one's nationality, and should not be used against them in immigration or citizenship petition decisions. In previous instances where relationship DNA testing was used to verify the place of origin, states assumed that a petitioner whose test results did not match their narrative were fraudulent and would deny admission. To use genetic test results (whether relationship DNA tests or biogeographic DNA tests) for migrant petitions in a way that is fair to the stateless individual will require a significant change in how states interpret DNA test results.

As DNA and genetic testing become more relevant in immigration and migrant proceeding, it is imperative to consider the unintended consequences of conflating genetic ancestry with nationality. It is imperative that both the state and stateless persons are protected. It is important that the logistics of DNA testing for nationality, whether relationship or ancestry, are considered so that costs are not a burden, and that personal information is handled with restrictions on use and confidentiality.
